# Stolen chemistry, rewritten genes

**DOI:** 10.1093/plphys/kiag449

**Published:** 2026-06-27

**Authors:** Praveen Khatri

**Affiliations:** Assistant Features Editor, Plant Physiology, American Society of Plant Biologists, Rockville, United States; Department of Biology, University of Toronto at Mississauga, Mississauga, ON L5L 1C6, Canada

Specialized metabolites play an important role in defining the chemical identity of plant lineages. Closely related species often possess similar biosynthetic and metabolic capacities than distantly related species. Yet plant metabolism does not always follow this orderly pattern. Some metabolites appear sporadically across distant branches of the plant kingdom, creating a patchy distribution that is often attributed to convergent evolution (Umezawa 2003). Different plant lineages may have evolved the ability to make the same compound independently. However, convergence is not the only possible explanation. Genes can also be transferred horizontally across species boundaries and may carry new metabolic capacities with them (Mariault et al. 2025). Horizontal gene transfer (HGT) can be particularly interesting in the case of parasitic plants, for which close physical association with host tissues may provide the opportunity for transfer of genetic material between species.

Parasitic plants offer a particularly intriguing context for exploring this possibility. Dodders (*Cuscuta* spp.; Convolvulaceae, Solanales) are obligate stem parasites that attach to host plants through haustoria, specialized structures of the parasite's stem that connect it with host tissue (García et al. 2014; Costea et al. 2015). The parasite uses the haustoria to obtain water, nutrients, and other resources from the host. Haustoria also form cellular interfaces that allow the transport of macromolecules. Previous studies have shown that parasitic plants can acquire host genes through HGT (Zhang et al. 2014; Yang et al. 2016). However, the mechanism by which such acquired genes become functional parts of the recipient genome is less clear.

In this issue of *Plant Physiology*, Ono et al. (2026) address how horizontally acquired genes can become functional components of specialized metabolism through investigating the occurrence of sesamin in dodders (*Cuscuta* spp.; Convolvulaceae, Solanales). Sesamin is a phenylpropanoid-derived specialized lignan metabolite best known from sesame (*Sesamum indicum*; Pedaliaceae, Lamiales) and related Lamiales species (Umezawa 2003). In sesame, the biosynthesis of sesamin depends on a cytochrome P450, CYP81Q1, also known as piperitol/sesamin synthase (PSS), which converts pinoresinol into piperitol and then into sesamin (Ono et al. 2006). Pinoresinol, the precursor of sesamin, is widespread across land plants, whereas the accumulation of sesamin is rather limited. To date, *Cuscuta* is the only genus in Convolvulaceae known to accumulate sesamin (Umezawa 2003; He et al. 2010). This limited taxonomic distribution raises the question of whether dodders independently evolved sesamin biosynthesis or acquired the enzyme from another plant lineage. Ono et al. (2026) demonstrate that, during evolution, *Cuscuta* acquired a functional metabolic gene, retained its catalytic activity, and structurally remodeled it (Fig. 1).

**Figure 1:**
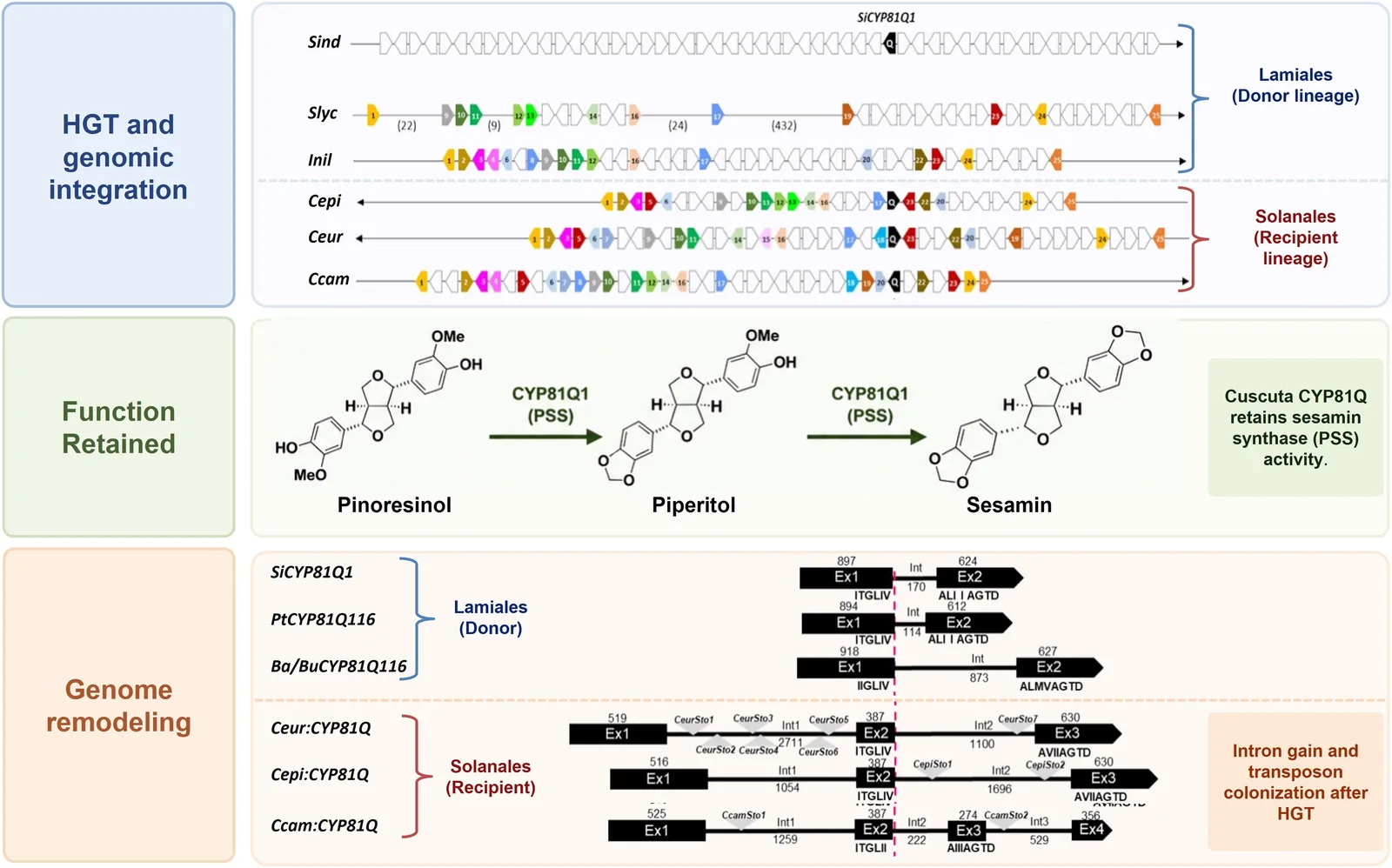
Parasitism-mediated acquisition and remodeling of *CYP81Q* in *Cuscuta*. A simplified model summarizing how a horizontally transferred *CYP81Q/PSS* gene became associated with sesamin biosynthesis in *Cuscuta*. The top panel depicts the comparative genomic context of *CYP81Q* genes, showing that *Cuscuta CYP81Q* genes lie within a Solanales-like genomic context, whereas *CYP81Q* sequence identity is highest with Lamiales *CYP81Q* genes. Colored gene symbols indicate conserved neighboring genes used for synteny comparison, and black symbols labeled Q indicate *CYP81Q* genes. The middle panel shows that the catalytic activity of *CYP81Q/PSS* is retained, converting pinoresinol to piperitol and then to sesamin. The bottom panel provides an overview of post-transfer structural remodeling of *CYP81Q* genes, in which *Cuscuta* genes show intron gain and transposon colonization relative to simpler Lamiales *CYP81Q* gene structures. Black boxes represent exons, connecting lines represent introns, gray triangles indicate transposons. Small black triangles indicate transposon remnants. The pink dashed line marks the conserved ancestral intron position shared between Lamiales *CYP81Q* genes and *Cuscuta CYP81Q* genes. Together, these observations support a model in which *Cuscuta* acquired *CYP81Q* through parasitism-mediated horizontal gene transfer, retained sesamin synthase activity, and subsequently remodeled the acquired gene within the recipient genome. Adapted from Ono et al. (2026).

Ono et al. (2026) first showed that the accumulation of sesamin in *Cuscuta* reflects de novo biosynthesis rather than simple uptake from host plants. The authors found sesamin in several species of *Cuscuta* and identified *Cuscuta* genes that were similar to sesame CYP81Q1. These proteins also possess the characteristic amino acid residues expected for sesamin synthase activity. The activity of *Cuscuta* CYP81Q proteins was confirmed by functional in vitro enzyme assays: several *Cuscuta* CYP81Q proteins were able to convert pinoresinol into sesamin via the intermediate piperitol. Together, these results supported the biochemical capacity of *Cuscuta* to produce sesamin.

Further evolutionary analyses placed this biochemical result in a broader genomic context. Phylogenetically, *Cuscuta* CYP81Q genes clustered with the CYP81Q genes of Lamiales species that contain sesamin, and not in the cluster of CYP81-related genes from Solanales species. Nor were CYP81Q genes detected in *Ipomoea*, the closest nonparasitic relative of *Cuscuta*. Another piece of evidence was provided by synteny, an analysis of the conservation of neighboring genes. *Cuscuta* CYP81Q genes are embedded within a genomic region whose neighboring genes are conserved among Solanales species, including *Cuscuta*, *Ipomoea*, and tomato (*Solanum lycopersicum*), but this Solanales-like genomic block is not conserved around CYP81Q genes in Lamiales species such as sesame. Thus, the gene itself has a Lamiales-like evolutionary identity, whereas its genomic neighborhood has a Solanales-like structure. This combination supports a model in which CYP81Q was horizontally inserted into an ancestral *Cuscuta* genome after divergence from *Ipomoea* and before the diversification of major *Cuscuta* lineages, followed by vertical inheritance and lineage-specific diversification.

Ono et al. (2026) also reported a plausible biological context for gene movement and then examined the structural fate of the transferred gene. Cuscuta *campestris* can parasitize sesame, and parasitism induces sesame CYP81Q1 expression. The authors detected both fully spliced and intron-retained CYP81Q1 transcripts in sesame, and intron-retained CYP81Q1 RNA was also detected in *Cuscuta* tissue near the parasitic interface. These observations suggest that host transcripts may move into the parasite during parasitism and could provide material for HGT, although the molecular steps that would convert a transferred RNA into a stably inherited parasite gene remain unresolved. After acquisition, the transferred CYP81Q gene did not remain structurally static. Lamiales CYP81Q genes have a relatively simple exon-intron structure, whereas *Cuscuta* CYP81Q genes are intron rich. Introns are noncoding gene segments that are removed from RNA before translation, and many of the newly acquired introns in *Cuscuta* contain transposable elements, or mobile DNA sequences, and their remnants. Importantly, the transposable elements associated with these introns appear to be endogenous *Cuscuta* elements rather than host-derived elements, suggesting that the acquired CYP81Q gene was remodeled by the recipient genome after HGT. This remodeling was not limited to CYP81Q: predicted HGT-derived genes in *C. campestris* show increased intron numbers compared with their predicted donor orthologs, whereas comparable non-HGT gene pairs do not show the same pattern. This broader analysis suggests that intron gain may be a recurring feature of retained HGT genes in *Cuscuta*. The results should still be interpreted carefully, because the authors did not demonstrate that transposable elements directly created each new intron. However, the repeated association between intron gain and mobile-element signatures suggests that transposons contributed to post-transfer gene remodeling. Yet successful HGT requires more than genomic accommodation; the acquired enzyme also needs a metabolic context in which it can be useful.

In *Cuscuta*, metabolic context may also have helped maintain CYP81Q function. Pinoresinol, the precursor of sesamin, can also be shunted into other lignan pathways by the enzyme pinoresinol/lariciresinol reductase (PLR). Ono et al. (2026) revealed that PLR-like genes are not found in *Cuscuta*, but they are found in other related non-parasitic species. This absence may have reduced competition for the pinoresinol substrate and thus allowed the flux to be channeled toward sesamin and other lignans via the acquired PSS enzyme (Kim et al. 2009; Murata et al. 2015). In this sense, HGT supplied the catalytic tool, but the existing metabolic landscape of the parasite may have helped determine whether that tool became useful.

These findings make *Cuscuta* CYP81Q more than an example of a borrowed enzyme. The gene crossed a major phylogenetic boundary, retained specialized catalytic activity, and became embedded in a different genomic and metabolic environment. At the same time, *Cuscuta* CYP81Q has gone through structural changes of introns and colonization by transposons, while retaining enzyme function. This combination links three processes that are often discussed separately: parasitism, horizontal gene transfer, and specialized metabolic innovation.

There are several questions still to be answered. The route from which a mobile host transcript is inherited into the parasite genome has not yet been determined. The relationship between transposable elements and intron gain also needs further testing, because association does not by itself establish causality. Finally, the biological role(s) of sesamin and related lignans in dodders remain an open question. Do these compounds play a role in the development of the parasite, seed survival, host interactions, or defense? Ono et al. (2026) place *Cuscuta*'s sesamin production in an evolutionary and biochemical context and present it as a model to understand how metabolic genes are acquired in different plant genomes.

**Recent related articles published in *Plant Physiology***

- Zangishei et al. (2022) demonstrated that the small RNA landscapes of parasitic plants are dynamic and include parasite-specific retention, loss and gain of miRNAs. Their analysis of *Cuscuta campestris* and *Orobanche aegyptiaca* has also revealed the presence of *Cuscuta* microRNA which seems to be the result of horizontal gene transfer, suggesting that parasitism can drive regulatory and genomic innovation.
- Fichman et al. (2024) showed that *Cuscuta* can serve as a bridge for rapid plant to plant communication transmitting reactive oxygen species, calcium and membrane-potential signals across host-parasite network, providing a model of inter-plant molecular communication, in addition to nutrient exchange, via haustorial connections.

## Data Availability

No data is generated in this study.

## References

[kiag449-B1] Costea M, García M, Baute K, Stefanović S. 2015. Entangled evolutionary history of *Cuscuta pentagona* clade: a story involving hybridization and Darwin in the Galapagos. Taxon. 64:1225–1242. 10.12705/646.7.

[kiag449-B2] Fichman Y et al 2024. Rapid plant-to-plant systemic signaling via a *Cuscuta* bridge. Plant Physiol. 196:716–721. 10.1093/plphys/kiae339. 38888995 PMC11483505

[kiag449-B3] García MA, Costea M, Kuzmina M, Stefanović S. 2014. Phylogeny, character evolution, and biogeography of *Cuscuta* (dodders; Convolvulaceae) inferred from coding plastid and nuclear sequences. Am J Bot. 101:670–690. 10.3732/ajb.1300449. 24688058

[kiag449-B4] He XH et al 2010. Two new lignan glycosides from the seeds of *Cuscuta chinensis*. J Asian Nat Prod Res. 12:934–939. 10.1080/10286020.2010.506434. 21061214

[kiag449-B5] Kim HJ et al 2009. Metabolic engineering of lignan biosynthesis in *Forsythia* cell culture. Plant Cell Physiol. 50:2200–2209. 10.1093/pcp/pcp156. 19887541

[kiag449-B6] Mariault L, Puginier C, Keller J, El Baidouri M, Delaux PM. 2025. Mechanisms, detection, and impact of horizontal gene transfer in plant functional evolution. Plant Cell. 37:koaf195. 10.1093/plcell/koaf195. 40834228 PMC12448838

[kiag449-B7] Murata J, Matsumoto E, Morimoto K, Koyama T, Satake H. 2015. Metabolic engineering of lignan biosynthesis in *Forsythia* cell culture. PLoS One. 10:e0144519. 10.1371/journal.pone.0144519. 26641084 PMC4671638

[kiag449-B8] Ono E et al 2006. Formation of two methylenedioxy bridges by a *Sesamum* CYP81Q protein yielding a furofuran lignan, (+)-sesamin. Proc Natl Acad Sci U S A. 103:10116–10121. 10.1073/pnas.0603865103. 16785429 PMC1502515

[kiag449-B9] Ono E et al 2026. Transposon-colonized intron gain follows parasitism-mediated horizontal transfer of a cytochrome P450 gene. Plant Physiol. 201:kiag335. 10.1093/plphys/kiag335. 42378117 PMC13591195

[kiag449-B10] Umezawa T . 2003. Phylogenetic distribution of lignan producing plants. Wood Res. 90:27–110. http://hdl.handle.net/2433/53098.

[kiag449-B11] Yang Z et al 2016. Horizontal gene transfer is more frequent with increased heterotrophy and contributes to parasite adaptation. Proc Natl Acad Sci U S A. 113:E7010–E7019. 10.1073/pnas.1608765113. 27791104 PMC5111717

[kiag449-B12] Zangishei Z et al 2022. Parasitic plant small RNA analyses unveil parasite-specific signatures of microRNA retention, loss, and gain. Plant Physiol. 190:1242–1259. 10.1093/plphys/kiac331. 35861439 PMC9516757

[kiag449-B13] Zhang D et al 2014. Root parasitic plant *Orobanche aegyptiaca* and shoot parasitic plant *Cuscuta australis* obtained Brassicaceae-specific strictosidine synthase-like genes by horizontal gene transfer. BMC Plant Biol. 14:19. 10.1186/1471-2229-14-19. 24411025 PMC3893544

